# B cell depletion in diffuse progressive systemic sclerosis: safety, skin score modification and IL-6 modulation in an up to thirty-six months follow-up open-label trial

**DOI:** 10.1186/ar2965

**Published:** 2010-03-25

**Authors:** Silvia Bosello, Maria De Santis, Gina Lama, Cristina Spanò, Cristiana Angelucci, Barbara Tolusso, Gigliola Sica, Gianfranco Ferraccioli

**Affiliations:** 1 Division of Rheumatology, Catholic University, Medical School, Via G. Moscati, 31 - Rome, 00168, Italy; 2 Institute of Histology and Embryology, Catholic University, Medical School, L.go F.Vito, 1 - Rome, 00168, Italy

## Abstract

**Introduction:**

An over-expression of CD19 has been shown in B cells of systemic sclerosis (SSc) and B cells are thought to contribute to the induction of skin fibrosis in the tight skin mouse model. The aim was to define the outcome on safety and the change in skin score after rituximab therapy in SSc patients and to correlate the clinical characteristics with the levels of interleukin (IL)-6 and with the immune cell infiltrate detected by immunohistochemistry.

**Methods:**

Nine patients with SSc with mean age 40.9 ± 11.1 years were treated with anti-CD20, 1 g at time 0 and after 14 days. Skin biopsy was performed at baseline and during the follow-up. B-cell activating factor (BAFF) and IL-6 levels were also determined at the follow-up times.

**Results:**

After 6 months patients presented a median decrease of the skin score of 43.3% (range 21.1-64.0%), and a decrease in disease activity index and disease severity index. IL-6 levels decreased permanently during the follow up. After treatment, a complete depletion of peripheral blood B cells was observed in all but 2 patients. Only 3 patients presented CD20 positive cells in the biopsy of the involved skin at baseline.

**Conclusions:**

Anti-CD20 treatment has been well tolerated and SSc patients experienced an improvement of the skin score and of clinical symptoms. The clear fall in IL-6 levels could contribute to the skin fibrosis improvement, while the presence of B cells in the skin seems to be irrelevant with respect to the outcome after B cell depletion.

**Trial registration:**

ISRCTN77554566.

## Introduction

Although the pathogenesis of systemic sclerosis (SSc) remains unknown, the B cell abnormalities characterized by autoantibody production [1], hyper-γ-globulinemia and polyclonal B cell hyperactivity [2] are thought to play an important role in the disease. It has been previously described that SSc patients have distinct abnormalities of blood homeostasis and B cell compartments, characterized by expanded naïve cells and activated, but diminished, memory B cells [3]. Furthermore, the expression of CD19, a critical signal transduction molecule of B cells that regulates autoantibody production, is significantly increased in memory and naïve B cells in SSc patients [3,4]. Analysis of DNA microarrays of cutaneous biopsies from diffuse SSc (dSSc) patients demonstrated a higher expression of clusters of genes of CD20-positive cells [5].

In the tight-skin mice, a genetic model of human SSc, the CD19 signaling pathway appeared to be constitutively activated [6,7] and the loss of CD19 expression significantly up-regulated surface IgM expression, completely abrogated hyper-γ-globulinemia and autoantibody production, and also inhibited IL-6 production [7]. Additionally, in this animal model, the down-regulation of B cell function led to a decrease in skin fibrosis during the disease onset [8]. Likewise, in a bleomycin-induced SSc mouse model, another animal model that shares many characteristics with human SSc, CD19 deficiency inhibited the development of skin and lung fibrosis, hyper-γ-globulinemia, and autoantibody production [9]. Thus, B cells could have a relevant impact on the development of fibrotic changes as reported in the mouse scleroderma models [6-9] and also in CCl_4_-induced liver injury, in an antibody- and T cell-independent manner [10].

In several studies focusing on the pathogenesis of SSc, the increased levels of IL-6 in the skin, serum, and bronchoalveolar lavage fluid of SSc patients suggest a role of this cytokine in promoting fibrosis by enhancing inflammation [11-13]. Furthermore, immunohistochemistry data demonstrated an over-expression of IL-6 on endothelium and fibroblasts of involved skin of scleroderma patients compared with normal skin [14]. SSc dermal fibroblasts constitutively produce about a four-fold increase in IL-6 levels with respect to healthy controls fibroblasts [15] and secretion of IL-6 from lung fibroblast is induced by SSc lung-derived B cells [16]. Recently, it has been reported that B-cell activating factor (BAFF), an essential component of B cell homeostasis and a potent B-cell survival factor associated with autoimmune disease in humans, is increased in SSc patients compared with healthy controls [17]. In the tight-skin mice, BAFF antagonist augmented anti-fibrogenic cytokines and inhibited the development of skin fibrosis. Finally, after BAFF stimulation, B-cells had a significantly enhanced ability to produce IL-6 [18].

Two recent open-label studies reported the safety of anti-CD20 treatment in SSc patients; despite both studies describing a decrease in myofibroblast score on serial skin biopsies after treatment, only one reported an improvement in skin score [19,20]. In these two studies, lung function remained stable during follow up, whereas a case report suggested a possible beneficial role of rituximab on lung involvement in scleroderma disease [21].

The primary aim of the current prospective study was to evaluate the changes in the skin score from baseline to at least 6 up to 36 months of follow up after anti-CD20 therapy. Secondary aims were to assess the potential efficacy of rituximab on lung function, to investigate the modification in IL-6 and BAFF serum levels as biological parameters of disease activity, and to correlate the clinical characteristics with the immune cell infiltrate detected by immunohistochemistry.

## Materials and methods

### Patients and treatment

Nine patients with progressive cutaneous SSc involvement, who showed a worsening of skin score higher than 10% after the conventional cyclophosphamide therapy [22] (up to 6 g), were treated with rituximab, two infusions of 1000 mg, two weeks apart, together with 100 mg methylprednisolone at each infusion, after three months of wash-out. All patients fulfilled the American College of Rheumatology classification criteria for scleroderma [23] and gave their informed consent to enter the study, which was approved by our Ethics Institutional Committee. All patients accepted that their biographical and clinical information could be eventually published.

Inclusion criteria were: age older than 18 years, a worsening in skin score higher than 10% after the conventional cyclophosphamide therapy, and a diffuse disease with trunk involvement. Exclusion criteria were: rest dyspnoea or signs and symptoms of heart failure, serious and uncontrolled coexisting diseases, infection, immunodeficiency or a history of tuberculosis contact, or cancer. None of the patients was taking corticosteroids daily. Three patients were re-treated with rituximab 1 g × 2 (days 1 to 15): the first patient because after 18 months she presented with a reactivation of her arthritis, while the other two patients were re-treated after 12 months because they presented a precocious and quicker B cell-recovery at months 3 and 7 (CD19 >4.5%).

There were eight women and one man, with a mean (standard deviation (SD))age of 40.9 ± 11.1 years, and a median disease duration of 2.0 (range:1.0 to 12.0) years. Seven patients had an early disease, defined as a disease duration less than three years since the occurrence of Raynaud's phenomenon. All patients presented a diffuse skin disease (dSSc); moreover, six (66.7%) had antiScl70-Abs positivity and three (33.3%) only presented antinuclear antibodies (ANA) positivity (Table 1) [24]. All nine patients continued to receive iloprost (by an infusion of 0.5 to 2 ng/kg/minute for five days every two months), calcium-channel blockers (nifedipine 20 to 40 mg/day) and acetylsalicylic acid from the moment of medical diagnosis. One of the two patients with long disease also presented with a metacarpophalangeal and wrist arthritis and one patient had myositis with high creatine kinase levels. Both these patients received methotrexate 15 mg/week after cyclophosphamide, one for treatment of arthritis and the other for myositis therapy. Both patients experienced a worsening of their skin fibrosis despite this therapy.

**Table 1:** Demographic and clinical characteristics of nine patients treated with rituximab

**Age **(years) (mean (SD))	40.9 (11.1)
(median (range))	41.5 (21.0-55.0)
**Disease duration **(months) (mean (SD))	49.0 (73.1)
(median (range))	24.0 (12-240)

**Female **(number,%)	8 (88.9)
**Male **(number,%)	1 (11.1)

**ANA positivity **(number,%)	9 (100)
**Anti-Scl70 positivity **(number,%)	6 (66.7)

**Follow-up **(months)(mean (SD))	16.7 (12.6)
(median (range))	12 (6-36)

The values are indicated as the mean (SD), median (range) or percentage. ANA, antinuclear antibodies; SD, standard deviation.

The extent of skin involvement was evaluated by the Rodnan skin score, performed by two observers and their results averaged [25]. Every three months, activity index [26] and severity index were assessed [27] and Global Health Status (GH) and Health Assessment Questionnaire (HAQ) were administered to patients to evaluate the influence of the disease on daily functions. At the same time intervals, blood samples were collected to determine IL-6 and BAFF levels and to count CD19-positive cells by flow cytometry.

### Internal organ involvement

All nine patients underwent pulmonary function tests to define forced vital capacity (FVC) and diffusing capacity for carbon monoxide (DLCO) before treatment and every six months. High-resolution computed tomography (HRCT) was performed before treatment and every 12 months. Renal involvement was defined as a scleroderma crisis or the presence of proteinuria or elevation in creatinine serum level. Creatinine levels and urine analysis were performed every three months. Cardiac involvement was defined as the presence of conduction disturbance, left ventricular ejection fraction (LVEF) less than 50%, pulmonary artery systolic pressure (PASP) more than 35 mmHg or presence of myocarditis; electrocardiography (ECG) and echocardiography were performed at the beginning of the treatment and every six months. Gastrointestinal involvement was defined as the presence of gastro-esophageal reflux symptoms or the evidence of gastrointestinal motility disturbance by barium swallow performed before treatment.

### Biological marker detection

Serum levels of IL-6 and BAFF (R&D Systems, Minneapolis, MN, USA) were measured using an ELISA, as described by the manufacturer. Erythrocyte sedimentation rate, total immunoglobulin (Ig) G, IgM and IgA were part of the routine clinical care of each patient. ANA were determined by indirect immunofluorescence using Hep-2 cells as substrates and autoantibodies specificities were further assessed by ELISA (Shield, Dundee, UK). Peripheral blood CD19-positive cell count was obtained by flow cytometry every three months.

### Skin biopsies and immunohistochemical analysis

Skin biopsies were performed in seven patients, who gave their informed consent, before treatment and in the five patients that achieved 12 months of follow up from the beginning of anti-CD20 therapy. Four healthy controls gave their informed consent to undergo forearm skin biopsy. In dSSc patients, cutaneous specimens were taken from the distal forearm for the clinically involved skin and from the buttock for clinically uninvolved skin. The biopsies were fixed into 10% formalin for two hours followed by paraffin inclusion for histological and immunohistochemical analysis.

Immunohistochemistry was carried out on 5 μm thick sections on polylysine-coated slides. After routine deparaffinization and rehydration, antigen retrieval was performed. Slide-mounted sections were heated in a microwave oven at 700 watt twice for four minutes in 10 mmol/L sodium citrate buffer (pH 6.0). Tissue sections were allowed to cool at room temperature (RT). Quenching of endogenous peroxidase activity was performed with Tris-buffered saline (TBS; pH 7.6) containing 2% hydrogen peroxide for 10 minutes at RT. Blocking was performed with 20% normal goat serum in TBS for 60 minutes at RT.

The sections were incubated with anti-CD3 and anti-CD20 mouse monoclonal antibodies (mAbs, Clone PS1 and L26 respectively; IgG_2a_; Ylem, Rome, Italy) both 1:100 diluted in blocking solution (20% normal goat serum in TBS) for 60 minutes at RT. Then, the Super Picture Polymer detection kit (Zymed Laboratories, South San Francisco, CA, USA) was used for 30 minutes at RT. The chromogenic reaction was developed with 3,3'-diaminobenzidine tetrahydrochloride solution (Zymed Laboratories, South San Francisco, CA, USA). The nuclei were lightly counterstained with Mayer's hematoxylin. Negative controls without primary antibodies were performed for all reactions. As all mAbs were of IgG_2a _isotype, mouse mAb IgG_2a _served as an isotype-specific control. Human tonsil specimens were used as positive controls for both antibodies. All controls were run under the same conditions and the same IgG concentrations were used for the respective primary antibodies. Positive cells were counted by two independent observers in six randomly selected fields (total area: 7.38 mm^2^) for each section at × 400 magnification. Differences between observers about staining evaluation were resolved by consensus. The total number of positive cells was calculated.

### Statistical analysis

All analyses were carried out using SPSS 15.0 (Chicago, IL, USA). Categorical variables were expressed as numbers, and quantitative variables as mean ± SD if normally distributed, and as median plus range if not. Non-normally distributed data were compared using the Mann-Whitney's test, and the Wilcoxon's test for paired data. A value of *P *< 0.05 was considered statistically significant.

## Results

### Skin score, activity and severity indices

**Figure 1:**
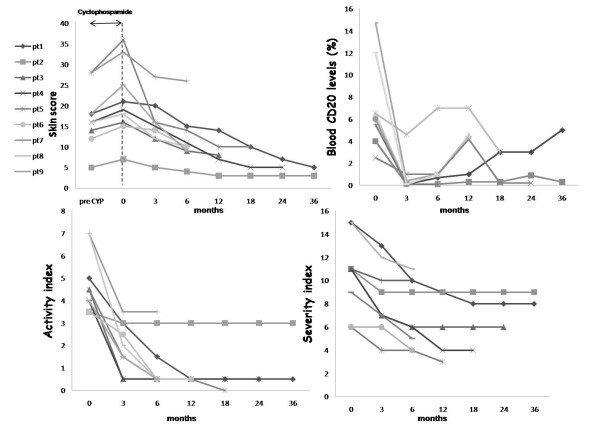
**Clinical improvement during follow up in nine systemic sclerosis patients treated with anti-CD20**. Clinical improvement in the nine patients treated with anti-CD20 during the follow-up times (3, 6, 12, 18, 24 and 36 months). Skin score, blood CD20 levels, severity index and activity index were assessed at baseline (0) and after 3, 6, 12, 18, 24 and 36 months. In the first graph precyclophosphamide (preCYP) skin score and skin score at time 0 (time of beginning of rituximab (RTX)) are reported. Each line represents the modification of different parameters in each patient during the follow up. Each symbol (on the left) represents one patient and corresponds to the number of the patient of Table 3.

All nine SSc patients treated with rituximab experienced an improvement of the skin score, activity index, severity index, HAQ and GH during the follow up if compared to pre-treatment values (Table 2 and Figure 1). Neither infections nor infusion reactions were observed. The only serious adverse event was the development of an occult breast cancer, which was thought to be unrelated to the study medication. The mean follow up was 16.7 ± 12.6 months: all patients reached a six-month follow up, five patients reached a 12-month follow up, four patients reached an 18-month follow up, three patients reached a 24-month follow up and two patients reached a 36-month follow up.

**Table 2:** Efficacy of rituximab on clinical and biologic parameters of nine SSc patients treated with anti-CD20 during the follow up

	Baseline	After 3 Months	After 6 Months	After 12 months*	After 18 months**	After 24 months***	After 36 months****
**Rodnan skin score**							
(mean (SD))	21.1 (9.0)	15.2 (6.0)	12.0 (6.1)	7.0 (4.0)	7.0 (3.5)	5.0 (2.0)	4.0 (1.4)
(median (range))	19.0 (7-36)	15.0 (5-27)	10.0 (4-26)	8.0 (3-14)	7.5 (3-10)	5.0 (3-7)	4.0 (3-5)
**Disease activity index**							
(mean (SD))	4.8 (1.3)	2.0 (1.2)	1.2 (1.2)	0.9 (1.0)	1.0 (1.3)	1.3 (1.4)	1.7 (1.8)
(median (range))	4.5 (3.5-7.0)	2.0 (0.5-3.5)	0.5 (0.5-3.5)	0.5 (0.5-3.0)	0.5 (0.5-3.0)	0.5 (0.5-3.0)	1.7 (0.5-3.0)
							
**Disease severity index**							
(mean (SD))	10.5 (3.2)	8.3 (2.9)	7.2 (2.8)	6.2 (2.8)	6.7 (2.2)	7.7 (1.5)	8.5 (0.7)
(median (range))	11.0 (6-15)	7.0 (4-13)	6.0 (4-11)	6.0 (3-9)	7.0 (4-9)	8.0 (6-9)	8.5 (8-9)
**HAQ**							
(mean (SD))	0.9 (0.7)	0.5 (0.5)	0.4 (0.5)	0.3 (0.7)	0.3 (0.6)	0.3 (0.6)	0.6 (0.6)
(median (range))	0.8 (0.1-2.4)	0.4 (0-1.6)	0.2 (0-1.5)	0 (0-1.5)	0 (0-1.2)	0 (0-1.1)	0 (0-1.3)
							
**GH **(mean (SD))	59.4 (20.9)	74.4 (16.5)	82.8 (16.6)	86.0 (10.8)	82.5 (21.8)	82.5 (17.7)	82.5 (17.7)
(median (range))	60.0 (30-85)	80.0 (50-95)	90.0 (50-95)	90.0 (70-95)	92.5 (50-95)	82.5 (70-95)	82.5 (70-95)
							
**Blood CD20%**							
(mean (SD))	6.8 (3.9)	0.7 (1.5)	1.7 (2.4)	3.0 (2.7)	2.0 (1.6)	1.0 (1.5)	3.0 (3.3)
(median (range))	6.0 (2.5-14.7)	0.1 (0.1-4.6)	1.0 (0.1-7.0)	4.0 (0.3-7.0)	2.0 (0.2-3.0)	1.0 (0.2-3.0)	3.0 (0.3-5.0)
							
**IgG mg/ml**							
(mean (SD))	1055 (233)	1001 (191)	1021 (158)	1028 (46.6)	946.2 (178)	944 (315)	951 (69)
(median (range))	1140 (729-1340)	932 (884-1440)	1030 (802-1220)	1005 (1000-1110)	938 (738-1170)	896 (656-1280)	951 (902-1000)
							
**IgA mg/ml**							
(mean (SD))	184.0 (44.4)	174.5 (39.2)	177.8 (63.5)	179.4 (69.8)	152.5 (59.9)	139.0 (67.5)	100.0 (24.0)
(median (range))	183.5 (119-249)	183.5 (119-262)	200.0 (75-262)	168.5 (93-281)	154.0 (79-222)	136.0 (73-208)	100.0 (83-117)
							
**IgM mg/ml**							
(mean (SD))	133.7 (21.7)	86.0 (12.8)	90.9 (28.9)	83.0 (18.7)	61.5 (6.7)	43.3 (10.6)	71.0 (1.4)
(median (range))	132.5 (95-157)	91.0 (56-136)	96.0 (40-136)	73.0 (64-105)	61.5 (54-69)	45.0 (32-53)	71.0 (70-72)
							
**BAFF pg/ml**							
(mean (SD))	1233.5 (683.3)	1719.4 (1264.3)	3257.8 (1571.8)	2057.0 (912.5)	2988.0 (1804)	3520.0 (1999)	3608.0 (2824)
(median (range))	875.6 (683-2601)	1008.6 (356-4038)	3141.8 (723-6682)	1580.6 (1321.6-3280)	2406.2 (1534-5605)	3224.8 (1684-5651)	3608.0 (1610-5605)
							
**IL6 pg/ml**							
(mean (SD))	3.7 (5.3)	1.0 (1.2)	0.6 (0.9)	0.4 (0.4)	1.2 (2.0)	0.1 (0.1)	0.1 (0.1)
(median (range))	1.7 (0.1-16.9)	0.1 (0.1-3.6)	0.1 (0.1-2.8)	0.4 (0.1-0.8)	0.25 (0.1-4.2	0.1 (0.1-0.1)	0.1 (0.1-0.1)

Clinical and biological parameters of nine SSc patients treated with anti-CD20 at baseline, after 3, 6, 12, 18, 24 and 36 months. The values are indicated as the mean (SD) and median (range). All patients had trunk skin involvement.

*Five SSc patients reached 12 months of follow up. **Four SSc patients reached 18 months of follow up. ***Three SSc patients reached 24 months of follow up. *****Two patients reached 36 months of follow up.

BAFF, B-cell activating factor; GH, Global Health Status; HAQ, Health Assessment Questionnarie; Ig, immunoglobulin; SD, standard deviation; SSc, systemic sclerosis.

Interestingly, in all nine patients treated with rituximab, the skin score improved gradually over time (Figure 1 and Table 2). After six months, the skin score improved in all the patients, decreasing from 21.1 ± 9.0 to 12.0 ± 6.1 (*P *= 0.001), with a median of improvement of 43.3% (range: 21.1 to 64.0%). Considering the last observation carried forward in each patient, the median skin improvement was 57.1% (range: 21.2 to 76.2).

After six months, the activity index decreased from 4.8 ± 1.3 to 1.2 ± 1.2 (*P *= 0.01) and the severity index from 10.5 ± 3.2 to 7.2 ± 2.8 (*P *= 0.01; Figure 1 and Table 2). All patients reported an improvement of their conditions as supported by the decrease in HAQ from 0.9 ± 0.7 to 0.4 ± 0.5 (*P *= 0.01) and an increase in GH from 59.4 ± 20.9 to 82.8 ± 16.6 (*P *= 0.01; Table 2). The only patient who did not present an improvement of the activity and severity indices, HAQ and GH, had a long disease duration.

### Organ involvement

The FVC and DLCO values showed no significant differences at follow up (96.8 ± 18.9% and 58.4 ± 14.2% of predicted value, respectively) compared with baseline (91.6 ± 20.7% and 58.0 ± 15.8% of the predicted value, respectively; *P *= ns for both comparison). Four (44.4%) patients presented an improvement higher than 10% of FVC, (median increase 14.9% (range: 11.8% to 29.5%)). None of the patients presented a reduction in FVC considered clinically significant (>10%), but one patient showed a decrease in FVC values suggesting a trend to a progression of her restrictive lung disease [28,29].

Two patients (22.2%) presented an isolated reduction of DLCO higher than 15%, both with an improvement in FVC values higher than 10% and with a stable echocardiography evaluation and no sign of pulmonary arterial hypertension. On the other hand, a clinical significant improvement in DLCO was reported in two patients (22.2%) [28,29] (Table 3).

**Table 3:** Demographic and clinical chracteristics of the study population

Patient	E/L	Disease duration (months)	Auto-antibodies	Follow-up duration (months)	FVC at baseline	FVC at the end of follow up	DLCO at baseline	DLCO at the end of follow up	Musculoskeletal involvement	Ulcers	Renal/cardiac/GI involvement	Concomitant medications
**1**	**E**	13	ANA	36	89%	104%	55%	54%	myositis	N	N/Y/Y	MTX
**2**	**L**	240	Scl70	36	101%	113%	52%	61%	arthritis	Y	N/N/Y	MTX
**3**	**E**	12	ANA	12	105%	96%	65%	69%	N	N	N/N/Y	-
**4**	**E**	24	Scl70	24	98%	101%	72%	65%	N	Y	N/N/Y	-
**5**	**E**	12	Scl70	18	100%	113%	56%	43%	N	Y	N/Y/Y	-
**6**	**E**	24	Scl70	6	95%	94%	86%	85%	N	Y	N/N/Y	-
**7**	**E**	24	ANA	6	44%	57%	54%	40%	N	Y	N/N/Y	-
**8**	**E**	32	SCL70	6	77%	78%	28%	47%	N	Y	N/Y/Y	-
**9**	**L**	60	SCL70	6	115%	115%	54%	62%	N	Y	N/N/Y	-

All patients had a diffuse cutaneous disease.

Renal involvement was defined as antecedent scleroderma crisis or the presence of proteinuria or elevation of creatinine serum level.

Cardiac involvement was defined as the presence of conduction disturbance, left ventricular ejection fraction less than 55%, pulmonary artery systolic pressure more than 35 mmHg or presence of myocarditis.

Gastro-intestinal involvement was defined as presence of gastro-oesophageal reflux symptoms or the evidence of gastrointestinal motility disturbance by barium swallow.

Concomitant medications: treatment other than iloprost by an infusion of 0.5 to 2 ng/Kg/minute, lasting six hours, for five days every two months, calcium-channel blockers (nifedipine 20 to 40 mg/day) and acetylsalicylic acid.

**Figure 2:**
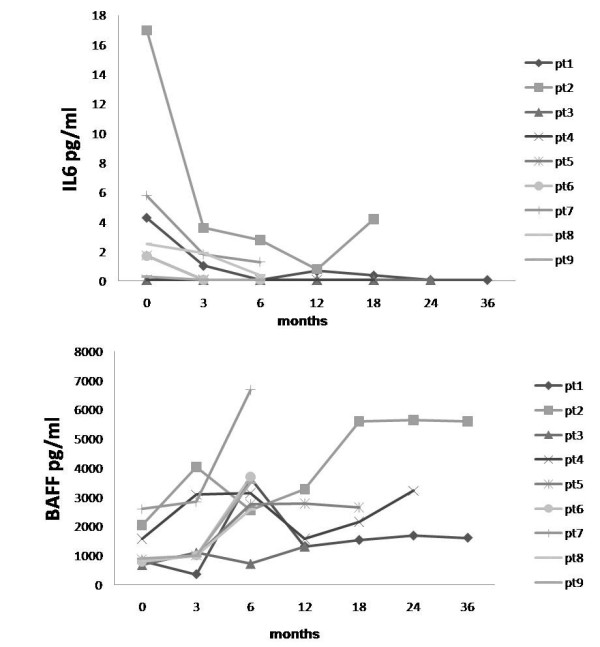
**(a) IL-6 and (b) BAFF levels at baseline and during follow up**. IL-6 and B-cell activating factor (BAFF) levels evaluated in nine patients treated with anti-CD20 at baseline and during the follow up. **(a and b) **Each line represents the modification of IL-6 and BAFF parameters in each patient during the follow up. Each symbol (on the right) represents one patient and corresponds to the number of the patient of Table 3.

The number, that identifies each patient, corresponds to the number of the patient in Figures 1 and 2.

ANA, antinuclear autoantibodies; DLCO, diffusing capacity for carbon monoxide; E, early disease (disease duration <3 years); FVC, forced vital capacity; GI, gastrointestinal; L, long disease (disease duration >3 years); MTX, methotrexate; N, no; Scl70, antitopoisomerasi I antibodies; Y, yes.

None of the patients showed signs of new or progressive cardiac disease, with stable ejection fractions and no modification on ECGs; none of the patients experienced renal crisis or symptoms suggesting progressive gastrointestinal disease.

### Biological markers

At baseline, patients presented high levels of IL-6 (3.7 ± 5.3 pg/ml), that permanently decreased after six months (0.6 ± 0.9 pg/ml, *P *= 0.02; Table 2 and Figure 2a). Three months after the rituximab infusion, circulating B cells evaluated by flow-cytometry were depleted (peripheral blood CD19 <0.1%) in all but one patient, and between 6 and 12 months they begun to repopulate. Upon B-cell depletion, BAFF levels increased relative to baseline (baseline: 1233.5 ± 683.3 pg/ml *vs *six months: 3257.8 ± 1571.8 pg/ml), while in one patient the BAFF levels did not increase until the time of repopulation (Figure 2b).

The autoantibody titers and IgG and IgA levels did not vary over the study period, while IgM levels decreased from 133.7 ± 21.7 mg/dl to 90.9 ± 28.9 mg/dl at six months follow up (*P *= 0.008), and to 83.0 ± 18.7 after 12 months of follow up (*P *= 0.04; Table 2).

Before rituximab treatment, one patient presented myositis with high creatine kinase levels, which decreased significantly after anti-CD20 treatment (data not shown). Creatine kinase levels remained within the normal range during the 36 months of follow up. The only patient with a long disease duration who did present the less significant clinical improvement, showed an important amelioration of her arthritis, with a change of disease activity score (DAS) from 4.3 to 2.0.

Three (42.9%) out of the seven patients, who underwent skin biopsies before treatment, presented CD20-positive cells on biopsies of the clinically involved skin and uninvolved skin; only one patient of these three repeated the biopsy after 12 months and it showed a depletion of dermal B cells. The other two patients were treated only for six months and they did not agree to a repeat biopsy.

**Figure 3:**
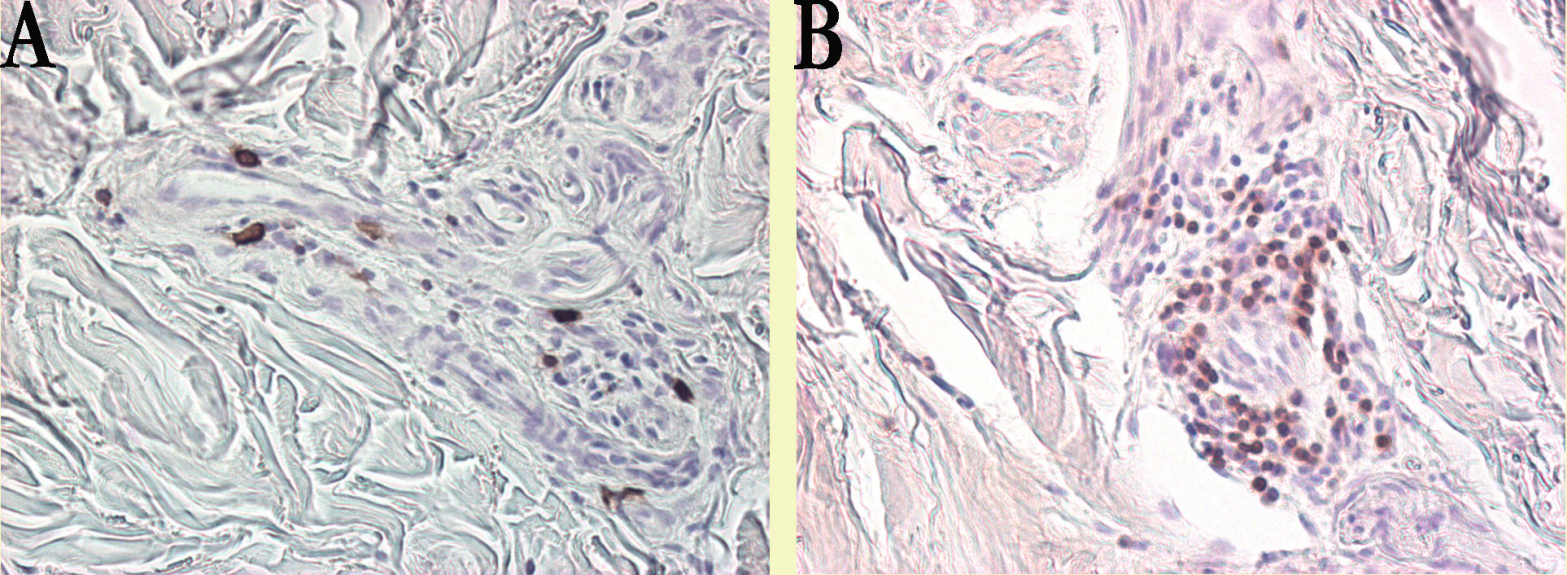
**B and T cell staining in systemic sclerosis biopsies**. In a forearm biopsy, immunohistochemistry revealed an expression of the B-cell marker CD20 in a limited number of **(a) **lymphocytes while a prominent expression of the T-cell marker CD3 was detected in the **(b) **perivascular lymphocytic infiltrate. Original magnification × 400.

CD3 lymphocytes were found, predominantly, in a perivascular location in the mid and deeper portion of the dermis in all the involved and uninvolved skin biopsies of patients before and after treatment with anti-CD20. Figure 3 illustrates the presence of B cells (a) and T cells (b) in forearm biopsy in patient number three before therapy. The mean number of CD3-positive cells in skin biopsies of four healthy subjects was 8.0 ± 2.0 and none presented B cells (data not shown). Before treatment, the mean number of CD3-positive cells was 54.7 ± 27.9 in involved skin and 65.6 ± 39.7 in uninvolved skin (*P *= ns; Figure 3). After treatment, a similar number of CD3-positive cells was found in involved skin (44.3 ± 24.0) and in uninvolved skin (62.7 ± 23.4) of the five patients who underwent skin biopsies after 12 months.

## Discussion

The results of our study suggest that B cell depletion in patients with early and progressive dSSc, leads to a clinically relevant decrease in skin involvement and to a stabilization of organ function. The only patient who showed a less clear-cut response either in terms of severity and activity indexes was the one with a long-standing disease.

In our study, the safety of anti-CD20 treatment in SSc patients was also confirmed in up to 36 months of follow up. The observed skin score improvement is more than expected as the spontaneous improvement in patients with similar disease, and comparable with the study by Smith and colleagues [19]. Recently, two studies assessed the safety of anti-CD20 treatment in scleroderma patients. In the first open-label trial, eight SSc patients experienced a skin score improvement up to 43% after 24 weeks from the beginning of anti-CD20 treatment [19], while in the second, a cohort of 15 SSc patients, with a follow up of 12 months, showed no improvement in the skin score [20]. Only the first group used the corticosteroids premedication.

In these two studies, all SSc patients, as in our study, had an early diffuse disease and patients were similar for age, disease duration and clinical characteristics [19,20]. It is interesting to note that despite little changes reported in the skin score after rituximab treatment in the largest cohort, a decrease in myofibroblast score was observed in several patients [20]. As the myofibroblast score correlates with the skin thickness score [20,30], these data suggest that a decrease in myofibroblast score could be a preclinical indicator of improvement of scleroderma skin fibrosis. Furthermore, Lafyatis and colleagues reported the presence of B cells in all but one skin specimen at baseline and a complete or nearly complete depletion of dermal B cells six months after administration of rituximab [20]. This suggests a biological effect on the skin after drug administration that could with new courses of the drug lead to a clinical skin improvement. In fact, we treated patients with a progressive cutaneous disease after conventional cyclophosphamide therapy. Moreover, we decided to re-treat two of our patients, because they presented a slower improvement of the skin score in the first six months of follow up and an earlier repopulation of B cells, similar to the data reported by the Lafyatis and colleagues, in which the majority of patients presented a precocious recovery of B cells between 6 and 12 months [20].

Interestingly, none of the SSc patients in the current study treated with anti-CD20 showed a progression of major end-organ involvement in a population with early diffuse disease that had a relatively high risk of organ complication. Parameters of internal organ involvement remained stable, but a further follow up in a more consistent group of patients is needed before drawing any conclusions.

The clear fall in IL-6 levels observed in our study is in agreement with findings obtained in a mouse model after B cell depletion [8]. This fall could be related, at least for the first stages, to the high dose of methylprednisolone used for the premedication in ours and the cohort of patients in the study by Smith and colleagues [19], but considering the follow times of evaluation (3 to 6 to 12 months) it has to be related to the rituximab treatment. This may suggest that IL-6 might contribute to the active phase of the disease. The decrease in IL-6 at the systemic levels could be the biological premise of the improvement in skin fibrosis. In fact, it has been previously reported that chronic IL-6 administration induces an increased synthesis of collagen in dermal fibroblasts [31] and in the liver [32]. Furthermore, IL-6 has been demonstrated to enhance resistance of lung fibroblasts to apoptosis, contributing to the fibrotic effect [33].

Immunohistochemistry clearly demonstrated the presence of T cells either in uninvolved or in involved skin, but B cells were seen only in some patients, as previously reported [5,19]. These data suggest that the most relevant contribution of B cells comes from the systemic pool. In fact, it appears clear that the response of skin fibrosis to B cell depletion does not rely on the presence of B cells in the skin, because most of our treated patients had no B cells, but very likely depends upon the general contribution to the autoimmune derangement given by the B cell compartments in lymphoid organs. B cells, with their multiple mechanisms as antibody-producing cells, antigen-presenting cells and profibrotic and proinflammatory cytokines producing cells (IL-6, IL-4, transforming growth factor-β), seem to be of great impact in the development of fibrosis. Thus, their modulation could inhibit skin fibrosis, as reported in the scleroderma mouse model [8], but the data on BAFF levels need to be interpreted since, as observed in patients with Sjogren's syndrome [34] or rheumatoid arthritis [35], the levels went up after B cell depletion.

## Conclusions

Our data suggest that anti-CD20 treatment is well tolerated and that dSSc patients experience an improvement of the skin score and of clinical symptoms. The clear fall in IL-6 levels may contribute to the skin fibrosis improvement, while the presence of B cells in the skin seems to be irrelevant with respect to the outcome after B cell depletion. Although we cannot draw any conclusion due to the limited number of cases, the response in the early disease patients was striking suggesting that a trial is warranted to confirm these preliminary data.

## Abbreviations

ANA: antinuclear antibodies; BAFF: B-cell activating factor; DAS: disease activity score; DLCO: diffusing capacity for carbon monoxide; dSSc: diffuse systemic sclerosis; ECG: electrocardiogram; ELISA: enzyme-linked immunosorbent assay; FVC: forced vital capacity; GH: Global Health Status; HAQ: Health Assessment Questionnaire; HRCT: high-resolution computed tomography; Ig: immunoglobulin; IL-6: interleukin-6; LVEF: left ventricular ejection fraction; mAbs: monoclonal antibodies; PASP: pulmonary artery systolic pressure; RT: room temperature; SD: standard deviation; SSc: systemic sclerosis; TBS: Tris-buffered saline.

## Competing interests

The authors declare that they have no competing interests.

## Authors' contributions

BS conceived and designed the study, collected data, performed the statistical analysis, interpreted and analysed data, and drafted the manuscript. MDS conceived and designed the study, collected data, interpreted and analysed data, and drafted the manuscript. LG carried out the immunohistochemistry, interpreted and analysed data, and revised the manuscript. SC carried out the immunohistochemistry, collected data, interpreted and analysed data, and revised the manuscript. AC carried out the immunohistochemistry, and collected data. TB carried out immunoassay and collected data. SG participated in the design of the study, analysed data and revised the manuscript. GF conceived and designed the study, interpreted and analysed data and drafted the manuscript. All authors read and approved the final manuscript.
